# Cytogenetic profile of chronic myeloid leukaemia patients resistant to imatinib at tertiary level in Indonesia

**DOI:** 10.1186/s13039-025-00737-0

**Published:** 2025-11-12

**Authors:** Ikhwan Rinaldi, Melva Louisa, Elly Yanah Arwanih, Farida Farah Adibah, Marcello Mikhael Kadharusman, Muhammad Alifian Remifta Putra, Kevin Winston, Yuli Maulidiya Shufiyani, Rafida Amalia Salma

**Affiliations:** 1 Division of Hematology and Medical Oncology, Department of Internal Medicine, Faculty of Medicine, Cipto Mangunkusumo National General Hospital, Universitas Indonesia, Jakarta, Indonesia; 2 Department of Pharmacology and Therapeutics, Faculty of Medicine, Universitas Indonesia, Jakarta, Indonesia; 3 Faculty of Medicine, Universitas Indonesia, Jakarta, Indonesia

**Keywords:** CML, Resistance, Cytogenetic, Cytogenetic abnormalities (ACAs)

## Abstract

**Background:**

Chronic Myeloid Leukemia (CML) is primarily driven by the Philadelphia chromosome, producing the BCR::ABL1 fusion protein. Although imatinib significantly improved CML outcomes, resistance remains a key challenge. Resistance often leads to cytogenetic abnormalities (ACAs), indicating poor disease prognosis. This is the first study that investigates genetic profiles in imatinib-resistant Indonesian CML patients.

**Method:**

The study included adult chronic-phase CML patients who met the criteria of treatment failure under the treatment of imatinib. Peripheral blood samples and bone marrow samples were collected and then processed for cytogenetic examination following the International System of Human Cytogenetic Nomenclature (ISCN) guidelines. BCR::ABL1 transcript levels were measured using Quantitative Real-Time PCR.

**Result:**

Out of 27 CML patients, the mean age was 39.15 years, with a male-to-female ratio of 1:1.5. The mean BCR::ABL1 international scale (IS) value was 37.20 ± 4.56, and patients on tyrosine kinase inhibitor (TKI) therapy had a median treatment duration of 80 months. Cytogenetic analysis showed Philadelphia chromosome (Ph) positivity in 11.11% of peripheral blood and 34.78% of bone marrow samples, with Ph negativity in 25.93% and 17.93%, respectively. Peripheral blood abnormalities included trisomy 8 (11.11%), additional Ph (7.41%), trisomy 19 (3.70%), and complex karyotypes (14.81%), while bone marrow abnormalities included trisomy 8 (13.04%), additional Ph (8.69%), trisomy 21 (4.35%), monosomy 7/7q- (8.70%), and complex karyotypes (43.45%).

**Conclusion:**

Cytogenetic anomalies such as trisomy 8, trisomy 19, and complex karyotypes may contribute to TKI resistance. Further study is needed to understand additional abnormalities observed.

## Background

Chronic Myeloid Leukemia (CML) is a myeloproliferative disorder characterized by the presence of the Philadelphia chromosome, which produces the BCR::ABL1 fusion protein [1, 2]. In 2017, it was estimated that there were 34,179 new cases of CML and 24,054 deaths globally. Even worse, the disease burden was especially higher in low sociodemographic regions, like Southeast Asia [3–5]. 

The introduction of imatinib marked a significant advancement in treating CML, contributing to improvements in patient outcomes [6]. However, imatinib resistance becomes a significant clinical hurdle, affecting approximately 10–30% of CML patients [7–10]. According to the ELN’s 2020 criteria, TKI resistance or “failure” response is defined as BCR::ABL1IS remains above 10% within 1–3 months if confirmed, above 10% at six months, above 1% at 12 months, or exceeds 1% at any point beyond 12 months of TKI treatment with resistance mutations or additional cytogenetic abnormalities [11]. 

The cytogenic hallmark of CML is the Philadelphia chromosome, t(9;22) (q34;q11), found in approximately 95% of CML cases [12]. TKI resistance leads to treatment failure and is often marked by the emergence of additional cytogenetic abnormalities (ACAs). For example, the presence of ACAs, like 3q26.2, has been associated with a poor prognosis and a higher frequency of ABL1 mutations, reinforcing TKI resistance [13]. Other chromosomal changes, such as an extra Philadelphia chromosome or an isochromosome of 17q, often indicate disease progression after imatinib treatment failure [14]. The emergence of ACAs during imatinib treatment may independently contribute to poorer survival outcomes [15]. 

Currently, no research has explored the cytogenic profile of imatinib-resistant CML patients in Indonesia. Therefore, this study aims to characterize the cytogenetic profiles of imatinib-resistant CML patients in Indonesia, examining localized genetic patterns and their association with resistance.

## Method

### Study participants

This cross-sectional study was conducted in Cipto Mangunkusumo Hospital and was approved by the Faculty of Medicine Universitas Indonesia Ethics Committee. The inclusion criteria were chronic-phase CML patients aged 18 years and above who met the “treatment failure” definition under imatinib therapy, based on the European LeukemiaNet (ELN) 2020 guidelines. Specifically, treatment failure was defined as a BCR::ABL1 International Scale (IS) remaining above 1% at 12 months or beyond. Accordingly, all samples in this study were collected at the point of confirmed imatinib failure, rather than at initial diagnosis. Exclusion criteria are individuals who meet treatment failure criteria using other tyrosine kinase inhibitors, chronic-CML patients with other neoplasms, and positive BCR::ABL1 non-CML patients. Subjects were collected through consecutive sampling over eight months. From the medical record, clinical data such as age, gender, hemoglobin count, leukocyte count, platelet count, BCR::ABL1 IS, and TKI therapy duration were collected. Peripheral blood (PB) and bone marrow (BM) samples were collected from patients for BCR::ABL1 levels and cytogenetics examination.

### Cytogenetic examination

#### Cell culture

The 3–5 mL heparinized whole blood specimens (BM & PB) were each transferred to culture tubes containing 10 ml of medium (RPMI 1640 with HEPES and 20% FBS). These tubes were placed in a CO2 incubator at 37 °C for 72 h.

#### Cell harvesting and chromosome preparation

Two and a half hours before completing the culture, 200 µl (10 µg/ml) of Colcemid solution was added to each culture tube. Subsequently, the cells were harvested by centrifugation at 1300 rpm for 10 min and washed three times with a potassium chloride solution (0.075 M). The cells were then fixed with acetic acid/methanol in a 1:3 ratio, using the standard air-dry method at room temperature for 3–5 days.

#### Chromosome staining and analysis

The Giemsa staining method (Perry and Wolff 1974) was employed to determine the number of mitotic divisions. Chromosomes were analyzed using Cytovision software. The chromosome numbers in cells in metaphase were counted (a minimum of 20 metaphases), and all karyotypes were documented using the ISCN (International System for Human Cytogenetic Nomenclature).

### BCR::ABL1 transcript level examination

The examination of BCR::ABL1 transcripts is conducted through Quantitative Real-Time PCR using the quantitative real-time PCR (qRT-PCR) method with the Xpert BCR-ABL Ultra kit (GXBCRABL-10 & GXBCRABL-US-10), following manufacturers’ protocol. The examination results are expressed as the BCR::ABL1/ABL1 ratio (International Scale).

## Results

A total of 27 patients were enrolled in the study. The characteristics of the research subject are shown in Table 1. The mean age of chronic-CML patients resistant to imatinib was 39 years, with 11 (40.7%) of the population being male and the remaining 16 (59.3%) female.

**Table 1:** Characteristics of research subjects

CML type	Values			
	All subjects (*n* = 27)	Bone marrow Ph (-) (*n* = 4)	Bone marrow Ph (+) (*n* = 8)	No metaphase (*n* = 11)
Age (mean)	39.15 ± 2.78	41.33 ± 4.49	36.50 ± 5.64	34.80 ± 3.95
**Gender**				
Male	11 (46.3%)	2 (50%)	5 (62.5%)	3 (27.27%)
Female	16 (59.3%)	2 (50%)	3 (37.5%)	8 (72.73%)
**Peripheral Blood**				
Hb (median (range))	8.5 (5.4–15.3)	8.8 (8.2–9.4)	7.6 (5.4–15.3)	9.3 (7.2–13.9)
Leukocytes (mean ± SD)	220,993 ± 35,214.50	240,783 ± 69,452.61	196,190 ± 64,439.76	231,791 ± 64,851.88
Platelets (mean ± SD)	542,050 ± 58,158.38	782,333 ± 106,163.92	344,250 ± 103,883.25	597,100 ± 75,735.57
BCR::ABL1 International Scale (mean ± SD)	37.20 ± 4.56	25.38 ± 12.08	58.29 ± 7.84	35.22 ± 6.04
TKI duration in Months (median (range))	80 (8–168)	30 (12–76)	46.5 (11–96)	96.5 (13–168)

Hb: hemoglobin; BCR::ABL1: breakpoint cluster region/Abelson murine leukemia viral oncogene homolog 1; SD: standard deviation

The bold texts indicate the main categories

The BCR::ABL1 ratio and the cytogenetic examination result are shown in Table 2. Out of the 27 patients recruited, four patients were rejected to undergo bone marrow sampling. From the remaining 23 bone marrow samples, no metaphase was discovered in 11 of the samples. Meanwhile, from 27 peripheral blood samples, no metaphase was found in 17 of the samples. Cytogenetic abnormalities were more frequently identified in bone marrow samples than in peripheral blood samples.

**Table 2:** BCR::ABL1 ratio and cytogenetic analysis result

No.	Specimen	BCR::ABL1 Examination results		Cytogenetic examination results	
		BCR::ABL1	International scale	Peripheral blood	Bone marrow
1	221214-1	57,90%	74,11%	46,X,-Y,+18[3]/46,XY,+12,15[1]/46,XY[12] 48,XY,+10,+18[1]	45,XY,−20[2]/45,XY, t(9;22)(q34:q11),−20[2]/ 45,XY,+12,rob(14;21)(q10:q10),−15,−21[2]/46,XY[9]
2	221214-3	35,89%	47,10%	45,XY,−20[5]/46,XY, del(20q)[5]/46,XY[10]	46,XY,−16,+18[1]/46,XY,+18,−19[2]/46,XY,+18,−19[3]/46,XY, t(9;22)(q34;q11)[4]/46,XY[12]
3	221221-1	41,15%	53,60%	45,XY,+8,−16,−19[4]/46,XY,−9,+18[2]/46,XY,−9,+mar[1]/46,XY[8]	45,XY,+8,−15,−21[3]/45,XY,+8,−9,+18,−19,−21[2]/46,XY, del(7q),+13,−16,+17,−21[1]/46,XY,+8,t(9;22)(q34;q11),−21[8]/46,XY[6]
4	221221-3	50,50%	64,64%	45,XX,−5,+6,t(9;22)(q34;q11),−16,del(20q)[4]/45,XX,+11,−20,−21[1]/46,XX,+6,−9[2]/46,XX[12]/49,XX,−5,+6,+8,−9,−10,+14,+17,+18,del(20q),+22[1]	42,X,-X,−8,t(9;22)(q34;q11),−10,−14,−19,del(20q),+21[1]/44,XX,+5,t(9;22)(q34;q11),- 15,−20,−21[1]/45,XX,+5,+6,−9,−15,−17[1]/46,XX,−9,+14,−16,+18[1]/46,X,-X,−7,t(9;22)(q34;q11),+14,−15,+18,del(20q),+21[2]/46,XX[9]
5	230201-1	41,15%	51,48%	46,XX,+6,−10[2]/46,XX,+18,−22[4]/46,XX[9]	46,X,-X, del(6q), t(9;22)(q34;q11),+10[1]/46,X,-X,+8,t(9;22)(q34;q11)[7]/46,XX[7]/47,X,-X,+8,t(9;22)(q34;q11),+16[2]
6	230,314	7,32%	8,28%	44,XY,−5,+6–8,−16[1]/45,XY,−20[5]/45,XY,−12[2]/46,XY,−5,+6,−7,+10[1]/46,XY,−10,+17[1]/46,XY,−5,+8[1]/46,XY[11]	44,XY,+8,−10,−15,−22[2]/46,XY,+8,−20[3]/46,XY[10]
7	220928-1	46,65%	65,31%	46,XY, del(5q)[2]/46,XY, t(9;22)(q34;q11)[8]/46,XY[9]/47,XY,+19[1]	No metaphase was found
8	221206-2	23,82%	29,88%	45,X,-X[3]/45,X,-X,+18,−21[2]//46,XX[11]	No metaphase was found
9	221019-4	54,08%	69,21%	45,XX,−7[2]/45,XX,−18[2]/46,XX, t(9;22)(q34;q11)[7]/46,XX[6]	Sample wasn’t collected
10	221012-3	27,31%	34,04%	45,XX,−16,−22[3]/45,XX,−22[2]/44,X,-X,−10[2]/46,XX[12]	Sample wasn’t collected
11	221012-1	31,30%	39,10%	No metaphase was found	42,XX,−5,−8,−9,−12[1]/45,XX,−22[2]/45,X,-X,+11,−22[1]/46,XX,+6,−11[2]/46,XX, t(9;22)(q34;q11)[3]/46,Xx[11]
12	230104-2	14,77%	19,16%	No metaphase was found	42,X,-X,−16,−18,−20[5]/45,X,-X,+13,−20[3]/46,XX[12]
13	230104-3	23,82%	29,74%	No metaphase was found	45,XY,+6,t(9:22)(q34:q11),−15,−16,del (20q)[5]/46,XY, t(9:22)(q34:q11),−15,+18,del (20q)[5]/46,XY[8]/47,XY,+18,t(9:22)(34:q11),del(20q)[3]
14	230,124	33,52%	42,72%	No metaphase was found	44,Y,-X, t(9;22)(q34;q11),−20[6]/46,XY[13]/47,XY,+6,del(7q),+13,−20[1]
15	230201-2	6,51%	8,57%	No metaphase was found	43,XX,−11,rob(13;15)(q10;q10),−15,−19[2]/44,XX,−15[2]/46,XX,+18,−22[2]/46,XX[14]
16	230,215	43,79%	48,70%	No metaphase was found	43,X,-Y,+1,del(3q),−8,−9,−10,−12,+18[1]/43,XY,+3,del(3q),−8,−9,+11,−17,+18,−19,−20,−22[1]/44,XY,+6,−14,−20,−21[2]/45,XY,+18,−20,−22[2]/46,X,-Y,+3,del(3q),+11,−21[1]/46,XY[8]
17	221109-1	3,52%	4,62%	No metaphase was found	No metaphase was found
18	221,129	15,81%	19,69%	No metaphase was found	No metaphase was found
19	221206-1	44,05%	56,39%	No metaphase was found	No metaphase was found
20	221212-2	41,15%	51,78%	No metaphase was found	No metaphase was found
21	221214-2	27,31%	35,33%	No metaphase was found	No metaphase was found
22	221221-2	14,77%	19,54%	No metaphase was found	No metaphase was found
23	230,222	40,88%	44,24%	No metaphase was found	No metaphase was found
24	230,224	22,01%	24,40%	No metaphase was found	No metaphase was found
25	230,301	3,25%	36,38%	No metaphase was found	No metaphase was found
26	221,205	16,93%	21,58%	No metaphase was found	Sample wasn’t collected
27	221207-3	7,46%	9,34%	No metaphase was found	Sample wasn’t collected

The additional cytogenetic abnormalities (ACAs) observed in the study can be seen in Table 3. The definition for major route lesions and additional lesions followed the study by Clark et al. [15]. The label “other lesions” includes all other possible lesions that were discovered in the study but not included in the labels “original major route lesions” and “additional lesions.” Of the 23 bone marrow samples, four did not have Philadelphia Chromosome. Five bone marrow samples had ≥ 1 of the four major route lesions, and six peripheral blood samples had one of the major route lesions. There were 13 bone marrow samples with the additional lesions, while only four peripheral blood samples had complex karyotypes.

**Table 3:** Cytogenetic profile of samples

Cytogenetic profile	Peripheral blood (*n* = 27)		Bone marrow (*n* = 23)	
	Amount	Frequency (%)	Amount	Frequency (%)
**No Ph**	7	25.93	4	17.39
Loss of Y	1	3.70	1	4.35
**Original major route lesion**				
Trisomy 8	3	11.11	3	13.04
Additional Ph	2	7.41	2	8.69
Isochromosome 17q	0	0.00	0	0.00
Trisomy 19	1	3.70	0	0.00
**Additional lesions**				
Trisomy 21	0	0.00	1	4.35
3q26.2 lesion	0	0.00	0	0.00
Monosomy 7/7q-	0	0.00	2	8.70
11q21 lesion	0	0.00	0	0.00
Complex karyotype	4	14.81	10	43.45
**Other lesion**	10	37.04	12	52.17
**No metaphase**	17	62.96	11	47.83

The bold texts indicate the main categories

## Discussion

To our knowledge, this is the first study exploring the ACAs in chronic phase CML patients resistant to imatinib. Identifying ACAs is valuable as a higher frequency of ACAs, especially at a younger age in CML patients, is related to inferior response to TKI and survival [16, 17]. 

In CML, treatment failure or progression to accelerated or blast phase is often marked by the emergence of ACAs beyond the typical Philadelphia (Ph) translocation. In the Indonesian population, trisomy 8 and additional Ph translocation are the most common major route lesions. Additionally, complex karyotype, monosomy 7/7q−, and trisomy 21 are other common lesions identified. These ACAs are among the abnormalities that are recognized by current LeukemiaNet guidelines as raising the risk of disease progression. [11] The finding is similar to the German/Swiss CML4 trial and UK SPIRIT2 study, where the 9 ACAs reported are more commonly found in patients who are at high risk of progression [15, 18]. 

Although our study did not assess outcomes to subsequent lines of therapy, prior research has demonstrated that certain high-risk ACAs—such as isochromosome 17q, monosomy 7, and complex karyotypes—are associated with poorer responses even to second-line tyrosine kinase inhibitors (TKIs). This highlights the prognostic value of cytogenetic profiling beyond first-line failure [19]. However, due to the retrospective nature of this study and the limited availability of initial diagnostic karyotyping, it was not possible to ascertain whether the cytogenetic abnormalities observed were present at diagnosis or emerged during therapy. Nevertheless, several of the abnormalities detected, such as monosomy 7 and complex karyotypes, are recognized markers of disease acceleration and may suggest an evolving disease biology in imatinib-resistant patients [14]. 

Four samples of a Ph-negative CML were identified in the bone marrow sample. In the Ph-negative CML cases, the BCR::ABL1 fusion gene could be found in der(22) and/or der(9). A study highlighted that it is more commonly found on der(22), but another study showed that it is found commonly on der(9), and there was a case with dual fusion signals on both der(9) and der(22), mimicking the classic translocation pattern [20–22]. In this study, since there are no der(22) discovered, Ph-negative imatinib-resistant CML patients in Indonesia are mostly cases with rearrangement on der(9) or potentially dual fusion signal on both der(9) and der (22).

ACA might arise in Ph-negative CML during TKI treatment due to clonal cytogenetic evolution. The most common abnormalities are trisomy 8 and loss of the Y chromosome [15]. This is aligned with our study, where one Ph-negative CML (25%) was reported with trisomy 8 and another case (25%) with loss of the Y chromosome. However, it is worth noting that this study could not confirm whether the ACA was present at the initial diagnosis or acquired during the TKI treatment.

Studies exploring the prognostic significance of Ph-neg CML patients treated with TKIs are limited. A study by Luatti et al. demonstrated that most Ph-negative CML patients benefit from being treated with imatinib, and the treatment outcome is similar to Ph-positive CML patients [22]. Therefore, guidelines like ELN 2013 do not provide a warning to Ph-negative CML patients in the imatinib era [23]. 

Moreover, there is some discrepancy between the findings in peripheral blood and bone marrow samples. Similarly, a study by Berman et al. showed a significant discrepancy between findings in peripheral blood compared to bone marrow BCR::ABL1 transcript levels. All peripheral blood and bone marrow samples were collected concurrently; however, discrepancies in karyotype findings between the two likely reflect differences in mitotic activity, cell composition, and sample sensitivity. Moreover, some samples—especially from peripheral blood—failed to yield metaphases, likely due to low mitotic index, delayed processing, or suboptimal quality.Therefore, further studies are needed to support and conclude the discrepancy [24]. 

With the possible risk of the discrepancy between 2 samples, the European LeukemiaNet recommends a bone marrow aspirate as the preferred sample to undergo cytogenetic analysis, while peripheral blood may be used as an alternative when a bone marrow aspirate is not available [12]. Similarly, the American College of Medical Genetics and Genomics (ACMG) recommends bone marrow as the primary sample for cytogenetic analysis. Still, peripheral blood may be informative if the blast percentage is more than 10% [25].

Despite elevated BCR::ABL1 transcript levels, some patients had no detectable t(9;22) by conventional cytogenetics. This may be due to cryptic or variant rearrangements involving BCR::ABL1 that escape detection via karyotyping. In such cases, more sensitive techniques such as FISH or RT-PCR are required to confirm fusion events [26]. 

A limitation of this study is the low yield of metaphase cells in many samples, likely influenced by factors such as patient age, sample type (peripheral blood vs. bone marrow), and sample quality. Additionally, comparison data from imatinib-responsive patients were not available, which limits interpretation of prognostic relevance. Nonetheless, the study offers valuable insights into CML patients in Indonesia, which can guide future research and contribute to better treatment strategies for CML patients in the country. A follow-up study will further explore the epigenetic mutations and BCR::ABL1 point mutations to understand the mechanism of imatinib resistance in Indonesia.

## Conclusion

This is the first study exploring the ACAs in chronic phase CML patients that are resistant to imatinib in Indonesia. The common ACAs discovered are trisomy 8, additional Ph, complex karyotype, trisomy 21, and Monosomy 7/7q-, which are associated with an increased risk of disease progression. Identifying these ACAs at diagnosis would allow physicians to anticipate the risk of disease progression.

## Data Availability

No datasets were generated or analysed during the current study.
